# Rebound effect of hypothalamic-pituitary thyreotropic activity: a new model to better understand hypothyroidism

**DOI:** 10.1007/s40618-024-02480-6

**Published:** 2024-10-21

**Authors:** T. Piticchio, C. Luongo, P. Trimboli, D. Salvatore, F. Frasca

**Affiliations:** 1 Endocrinology Section, Department of Clinical and Experimental Medicine, Garibaldi Nesima Hospital, University of Catania, Catania, Italy; 2 Department of Medicine and Surgery, University of Enna “Kore”, Enna, Italy; 3 Department of Clinical Medicine and Surgery, University of Naples “Federico II”, Naples, Italy; 4 Clinic for Endocrinology and Diabetology, Lugano Regional Hospital, Ente Ospedaliero Cantonale, Bellinzona, Switzerland; 5 Faculty of Biomedical Sciences, Università della Svizzera Italiana (USI), Lugano, Switzerland; 6 Department of Public Health, University of Naples Federico II, Naples, Italy

**Keywords:** Thyroid, Hypothyroidism, TSH, Pituitary, Rebound, Thyreotropic activity

## Abstract

**Purpose:**

Hypothalamic-pituitary thyrotropic activity (HPta) is crucial since TSH is the mainstay for evaluating primary hypothyroidism (hT) and replacement therapy in clinical practice. Despite TSH values, some patients experience symptoms and metabolic alterations, raising several issues about hT. The aim of the study was to investigate factors influencing the TSH values achieved after a period of hT induced in a standardized and controlled manner (TSH_time1).

**Methods:**

Our institutional database was searched to extract records of differentiated thyroid cancer (DTC) patients undergoing a LT4 withdrawal protocol prior to radioiodine (RAI) administration. We collected clinical and biochemical parameters before LT4 discontinuation and after one month of induced hT. We performed Mann-Whitney U-test and linear regression analyses.

**Results:**

We included 102 patients, with a median age of 44 years. In univariate analyses, TSH_time1 was correlated with age (p 0.005) and the dose pro Kg/die of LT4 assumed until the discontinuation of LT4 (LT4_dose) (p 0.023). The higher the age, the lower the TSH_time1 level. The higher the LT4_dose, the higher the TSH_time1 level. After multivariate analysis, the fittest model included age, BMI, LT4_dose, and systemic inflammation response index with an adjusted R^2^ of 0.4.

**Conclusion:**

The study highlights new mechanisms that influence HPta. HPta progressively reduces with age, and this must be considered when evaluating TSH values in the elderly. Furthermore, we report for the first time a rebound effect of HPta, determined by the dose pro Kg/die of LT4 taken prior to its discontinuation. Inflammation and metabolic status also affect these phenomena.

**Supplementary Information:**

The online version contains supplementary material available at 10.1007/s40618-024-02480-6.

## Introduction

The hypothalamic–pituitary–thyroid (HPT) axis was described for the first time in the 20th century [1]. It regulates the secretion of thyroid hormones (THs) and the systemic availability of free-thyroxine (FT4) and free-triiodothyronine (FT3). Hence, it is crucial for systemic homeostasis, basal metabolic rate, and brain activity [2–4]. The HPT axis is mainly controlled by a negative feedback mechanism of circulating THs [Thyroid], thyrotropin (TSH) [Pituitary], and thyrotropin-releasing hormone (TRH) [Hypothalamus]. In addition, this system presents several interplays with the other hypothalamic-pituitary axes integrating central and peripheric inputs. Indeed, the functioning of HPT axis is necessary to adapt the synthesis and secretion of THs to several endogenous and exogenous factors, such as changes in ageing, body weight or body composition, thyroidal and non-thyroidal illness, pharmacologic therapies, and psycho-social stress [2, 5, 6]. Finally, central and peripheral deiodinases contribute also to the regulation of HPT axis [7]. In particular, selenodeiodinase 2 (D2), expressed in hypothalamus and pituitary, by activating 3,5,3’,5’-tetraiodothyronine (T4) to 3,5,3’-trioidothyronine (T3), seems to be the playmaker in the central regulation of TSH production [7].

Since TSH is considered the most reliable marker for assessing the severity of primary hypothyroidism (hT) [8], the activity of the HPT axis assumes a fundamental role in evaluating the adequacy of replacement therapy in patients with hT. However, despite the restoration of a normal TSH level by replacement therapy, some patients experience residual symptoms and metabolic abnormalities, which raises several issues regarding the ability to actually achieve euthyroidism, based on the simple normalisation of TSH levels [9–17]. Therefore, open questions on this topic are: (1) whether other biomarkers of thyroid homeostasis are available in addition to TSH [18, 19]; and (2) consequently, how to improve replacement therapy in patients with hT [9, 11, 13, 20].

To date, this issue has been studied mainly by focussing on the lower part of the HPT axis through the observation of TH levels and effects [10, 21–27]. Furthermore, in almost all studies on hT, the duration of this alteration and the speed with which it is established are not taken into account, with a potential confounding effect on the results. Therefore, more efforts are needed to better understand the factors that interfere with hypothalamic pituitary thyrotropic activity (HPta) in patients with hT, thus focussing on the upper part of the HPT axis.

An ideal model to investigate HPta should include thyroidectomized subjects who undergo a period of absolute withdrawal from thyroid hormone. This opportunity is provided by athyreotic patients diagnosed with differentiated thyroid cancer (DTC) who require radioiodine (RAI). These subjects are often in an excellent performance status (i.e., ECOG 0), hence otherwise healthy, and usually undergo a controlled and standardized period of hT by withdrawing levothyroxine (LT4) until the RAI administration.

The aim of the study was to investigate factors that influence HPta through TSH levels achieved by thyroidectomized DTC patients after a period of induced hypothyroidism in a standardized and controlled manner.

## Methods

### Study conduction

The study was conducted according to STrengthening the Reporting of OBservational studies in Epidemiology (STROBE) statement [28] The check list is reported as Supplemental Material.

The clinical setting in which the study was conducted and the selection of cases are very similar to those of another study by our research group that was already published [29]. However, for a better consultation of the same and a more comfort for the reader, we report them below.

### Institutional management of patients with thyroid carcinoma

Garibaldi Nesima Hospital is a high-volume tertiary care centre in Catania (Italy). In this metropolitan area, adult patients with DTC pathology report after total thyroidectomy are placed on a fixed dose of LT4 (i.e., 100 µgr/die) and then referred to the Department of Endocrinology of this hospital for subsequent treatments and long-term follow-up. According to institutional guidelines, DTC patients who are candidates for RAI administration continue to take 100 µgr/day of LT4 until withdrawal and receive I131 as soon as possible, within six months from total thyroidectomy. DTC patients assessed otherwise healthy are prepared to RAI by a standard protocol of hT induction: (1) LT4 therapy is stopped from 28 days before the day scheduled for RAI administration; (2) patients assume l-triiodothyronine at fixed daily dose during the first 14 days, while they do not assume thyroid hormones during the second 14 days. On the day of RAI administration, patients are hospitalized, individual anthropometric parameters are recorded, and laboratory tests are assessed including hemogram, TSH, FT4, and FT3 at institutional laboratory department.

### Case selection

Records of DTC patients treated with RAI at our institution between January 2016 and December 2019 were screened. Inclusion criteria were: (1) DTC healthy patients, prepared for RAI by withdrawing thyroid hormone replacement therapy; (2) Age > 18 years; (3) total thyroidectomy; (4) RAI performed within 6 months after surgery. Exclusion criteria were: (1) Age < 18 years; (2) concomitant treatments with corticosteroids or drugs with a known effect on HPta (i.e., phenothiazines, triciclic anti-depressant, glucocorticoids, dopamine, lithium, mitotane, CTLA4 blocking antibodies, tyrosine kinase inhibitors, EGF-R antagonists, and bexarotene) [30] or anticoagulation drugs; (3) concomitant major diseases potentially interfering with the parameters of the study (i.e., chronic inflammatory autoimmune diseases, acute or chronic infections, haematologic diseases, heart failure, atrial fibrillation, myeloproliferative disorders, hepatic or renal disorders, and endocrine disorders including diabetes mellitus).

### Laboratory measurements

Serum TSH, FT4, FT3 and thyroglobulin-Ab (TgAb) were measured using an immune enzyme assay platform called AxSYM (Abbott, Abbott Park, IL, USA). Serum thyroglobulin (Tg) was measured with a second generation chemiluminescent Tg immunoassay (Tg Access; Beckman Coulter, Brea, CA, USA).

### Data extraction

We extracted data collected just before the withdrawal of LT4 and data collected after a month of hT (just before RAI). Data collected just before the withdrawal of LT4: individual demographic and anthropometric parameters; dose of LT4 pro Kg/day taken from the day after thyroidectomy until withdrawal to induce hT (LT4_dose); TSH value (TSH_time0). Data collected after a month of hT: TSH (TSH_time1), FT3, FT4, thyroglobulin (Tg), anti-thyroglobulin antibodies (TgAb), creatinine, glycaemia, sodium, potassium, calcium, phosphorus, GOT, GPT, Gamma GT, and complete blood count.

### Measures

“Age” was considered as a categorical variable in a single analysis to evaluate the outcome across age groups, choosing as cut-off 65 years old (i.e., young < 65 years vs. old ≥ 65 years); in all the other evaluations “Age” was considered as continuous variable.

BMI was analysed as categorical variable for a better clinical comparison (i.e., BMI: 18.5-24.99 Kg/m^2^ - normal weight vs. BMI > 25 Kg/m^2^ - overweight/obesity).

Inflammatory status of patients at the end of hT period was expressed with Systemic Inflammation Response Index (SIRI), calculated as follow ([neutrophils x monocytes]/lymphocytes) from the complete blood count performed just before the RAI administration.

### Statistical analysis

The results were expressed as median values and interquartile range (IQR). A Mann Whitney U-test was performed to compare the differences between two independent samples. The correlations between variables were estimated using Pearson’s or Spearman’s correlation, as appropriate. Univariate regression analyses and a stepwise multivariate regression analysis were performed to evaluate which variables independently influence the TSH levels after induction of hT (i.e., TSH_time1).

A p-value < 0.05 was considered statistically significant. Statistical analyses were performed with the computer software Jamovi (version 2.3).

## Results

**Fig. 1:**
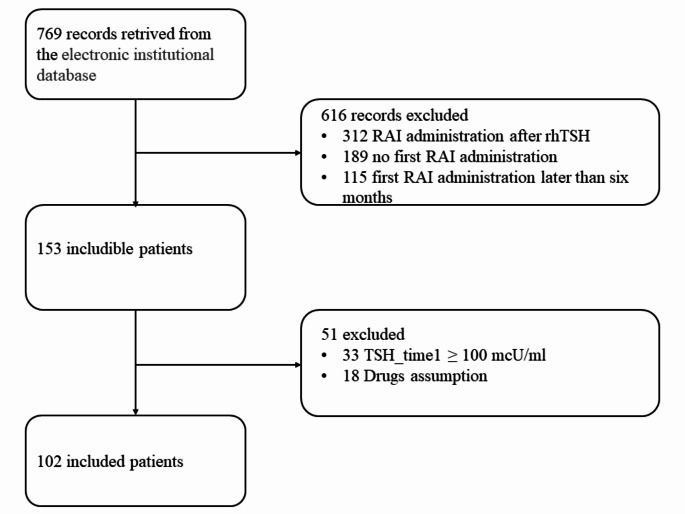
Flow diagram of case selection

Seven hundred sixty-nine records were retrieved from our electronic institutional database. According to the above selection criteria, 102 patients were included in the study (Fig. 1).

We selected 78 females and 24 males, 74 categorized as young and 28 as old, with overall median age and median BMI respectively of 44 years (35.5–55.5) and 27.1 Kg/m^2^ (23.7–31.7). Nine patients were smokers and 11 assumed levothyroxine before thyroidectomy. The median TSH_time0 was 1.84 µUI/ml (0.35–5.53) and the median TSH_Time1 was 55.6 µUI/ml (40.0-76.9). Finally, the median TSH increase was 72 folds (30–361). Table 1 illustrates the baseline characteristics of the study sample (Table 1).

**Table 1:** General parameters of the study sample

			Percentiles	
	*N*	Median	25th	75th
Age (years)	102	44.0	35.50	55.50
Weight (Kg)	102	75.0	63.0	84.0
BMI (Kg/m^2^)	102	27.11	23.725	31.65
LT4_dose (µgr/Kg/day)	102	1.504	1.33	1.72
TSH_time0 (µUI/ml)	102	1.84	0.35	5.53
TSH_time1 (µUI/ml)	102	55.6	40.0	76.9
TSH increase (Folds)	102	72.0	30.0	361
Creatinine (mg/dl)	102	0.90	0.81	1.05
Glucose (mg/dl)	102	86.0	77.75	97.25

**Fig. 2:**
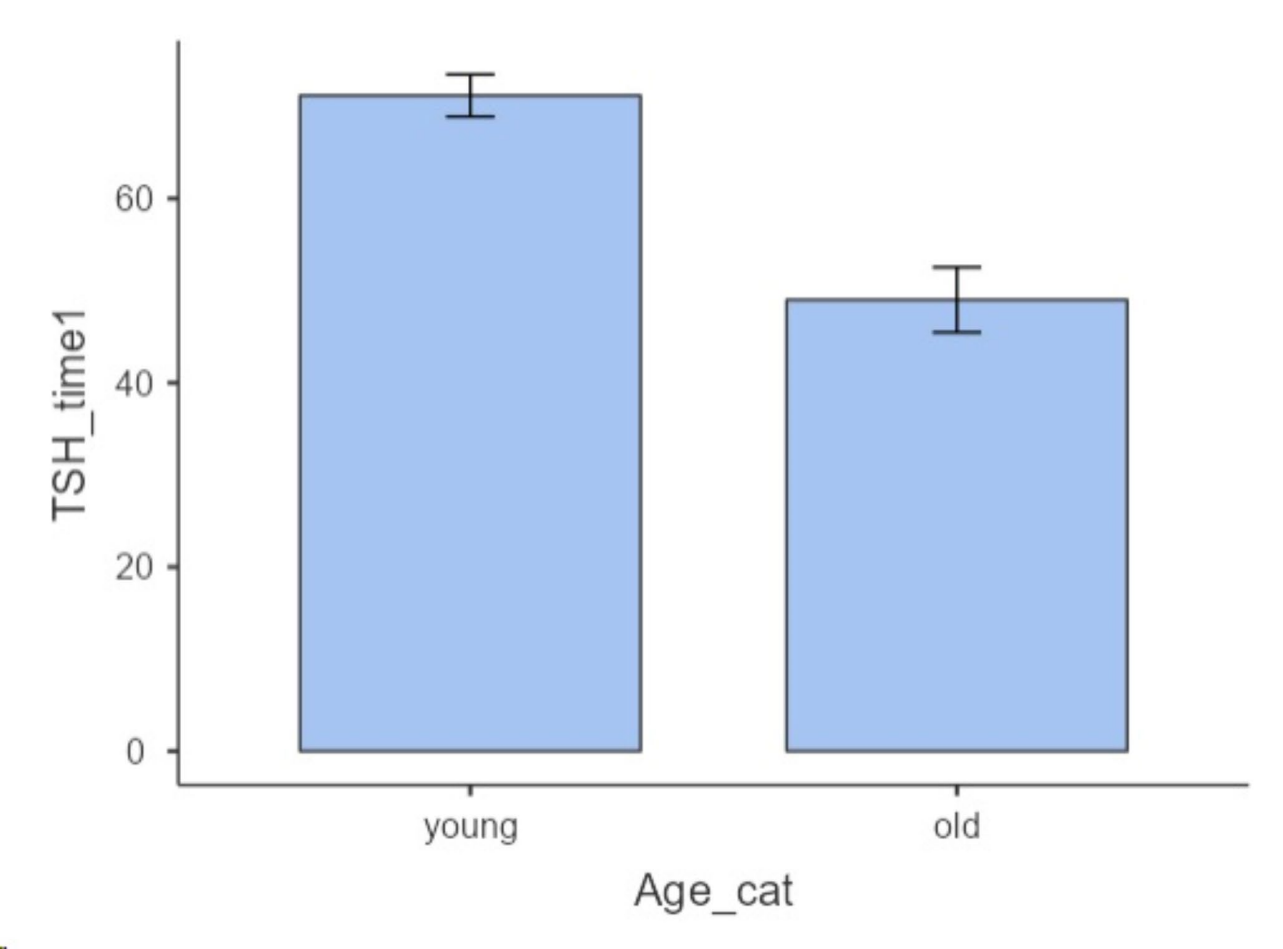
Comparison of TSH levels just before RAI between young and old patients

The Mann–Whitney U test showed that TSH_time1 levels (just before RAI) were significantly higher in young than older patients (p 0.006) (Fig. 2). By the other hand, TSH levels were not significantly different in males and females (p 0.959), normal and overweight/obese patients (p 0.591).

By univariate linear regression analysis, we explored independent factors (clinical features and biochemical parameters) affecting TSH levels during hT. We found that independent factors were age (p 0.005) and LT4_dose (taken from thyroidectomy until LT4 withdrawal) (p 0.023) (Table 2). By the other hand, sex, BMI_cat, TSH_time0, glucose, Tg, TgAb, SIRI, creatinine, smoking, and the other tested parameters (see Table 2) did not influence TSH_time1 levels. Multivariate Linear Regression analysis revealed that the best fit model included age (Effect: -0.52; 95%CI: -0.930.12; p 0.012), LT4_dose (Effect: 28.02; 95%CI: 10.04–46.0; p 0.003), BMI_cat (Effect: 13.74; 95%CI: -26.85– -0.63; p 0.040), and SIRI (Effect: -7.195; 95%CI: -15.42–1.03; p 0.055). Adjusted R^2^ showed that this model accounts for approximately 40% of the variance in the TSH_time1 levels (*p* < 0.001). Table 2 displays all parameters tested in univariate and multivariate analyses.

**Table 2:** Linear regression analysis to evaluate factors independently affecting TSH levels

	Univariate analyses			Multivariate analysis		
	Estimated Effect	95% CI	*p* value	Estimated Effect	95% CI	*p* value
Sex	-0.25	-10.0–9.52	0.959			
Age	-0.42	-0.71–0.13	**0.005**	-0.52	-0.93– -0.12	0.012
BMI_cat	2.48	-10.1–15.1	0.693	-13.74	-26.85– -0.6	0.04
TSH_time0	0.29	-0.12–0.70	0.161			
Smoking	-9.32	-15.9–1.43	0.091			
LT4 assumption prior to thyroidectomy	-7.38	-20.7–5.91	0.273			
Creatinine	8.17	-11.7–28.1	0.417			
Glucose	-0.11	-0.32–0.11	0.334			
Tg	0.01	-0.02–0.024	0.615			
TgAb	0.02	-0.01–0.049	0.284			
Sodium	-0.49	-2.76–1.78	0.668			
Potassium	-7.40	-20.1–5.25	0.248			
Calcium	2.69	-4.57–9.95	0.463			
Phosphorus	4.45	-4.79–13.9	0.337			
SIRI	-1.01	-5.94–3.92	0.685	-7.195	-15.42–1.03	0.055
LT4_dose	16.03	2.30–30.3	**0.023**	28.02	10.04–46.0	0.003
GOT	-0.09	-0.31–0.14	0.445			
GPT	-0.01	-0.18–0.15	0.871			
Gamma GT	-0.09	-0.34–0.15	0.448			

**Fig. 3:**
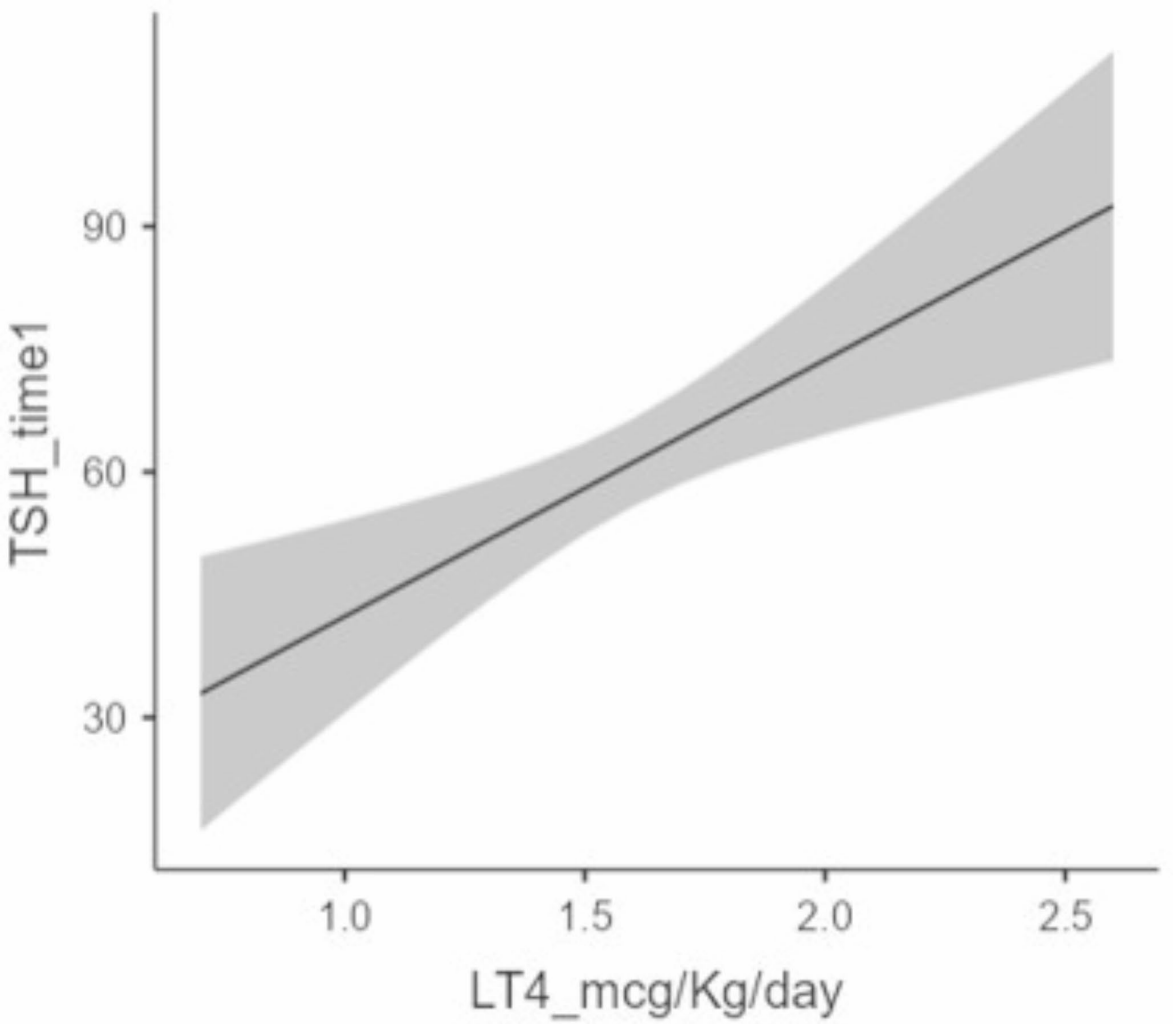
Graphic representation of the relationship between TSH levels the last day of hT and the LT4_dose taken from post-thyroidectomy until starting withdrawal by multivariate regression analysis

**Fig. 4:**
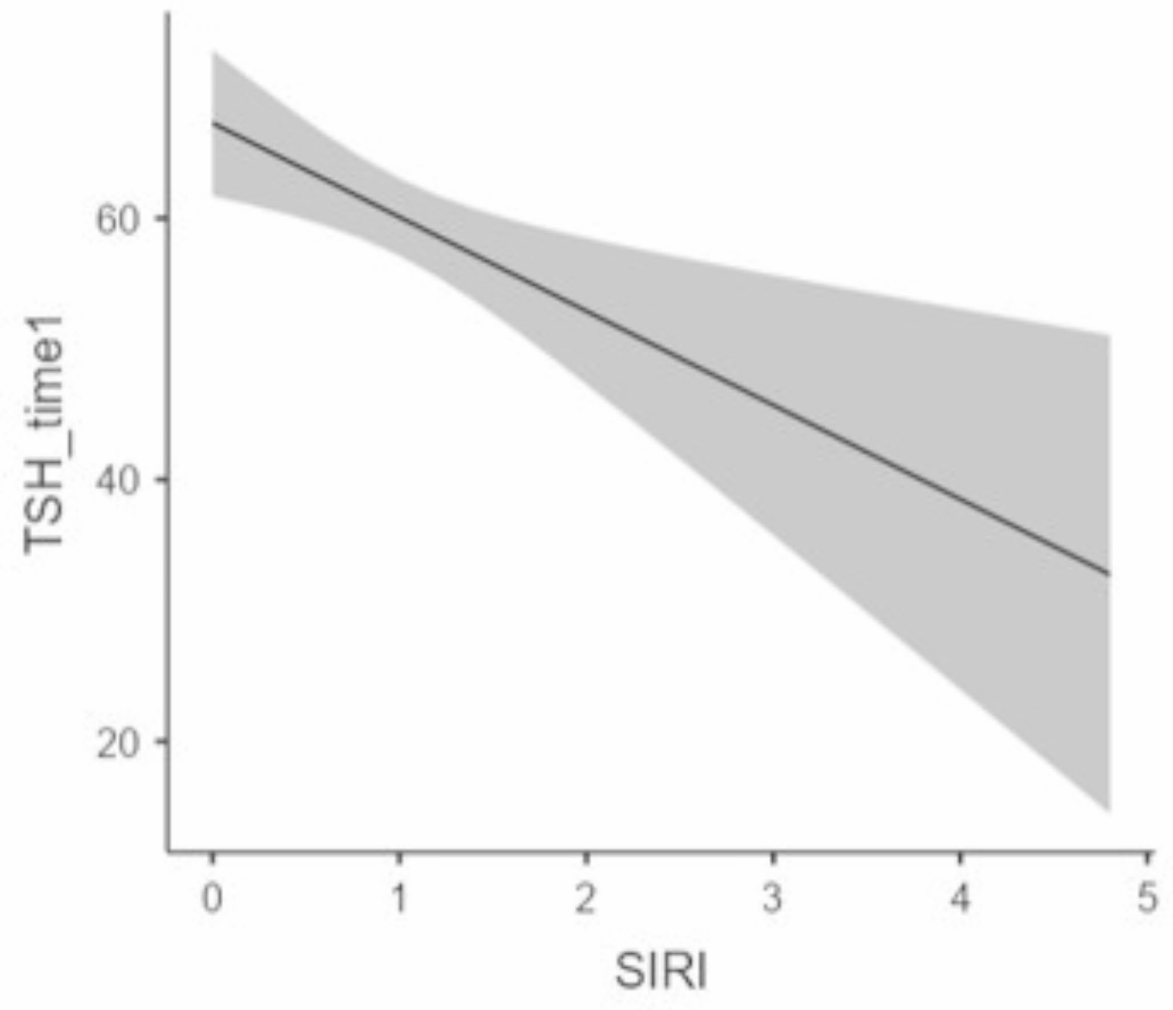
Graphic representation of the relationship between TSH and SIRI levels the last day of hT by multivariate regression analysis

Figures 3 and 4 show how the dose of LT4 and inflammation affect HPta. The higher the level of LT4 (µgr proKg/die) taken after thyroidectomy until the beginning of hT induction, the higher the level of TSH achieved at the end of hT. The higher the patient’s inflammation (SIRI level), the lower the TSH level achieved at the end of hT. Furthermore, the BMI ≥ 25 Kg/m^2^ and the increasing age resulted in a lower production of thyrotropin by the hypothalamic pituitary unit. Finally, we excluded eventual correlations of TSH_time0 levels with patient’s age (Spearman’s ρ: -0.056; p: 0.6) or with LT4_dose (Spearman’s ρ: -0.188; p: 0.093).

## Discussion

The first evidence from the study was the strong role of ageing in HPta regulation. The higher the age, the lower the TSH value achieved just before RAI, suggesting that ageing reduces HPta. These results agree with previous evidence and confirms the reliability of our series [31–34]. In this respect, Luongo et al. hypothesised that an age-related decline of TSH production in response to hT could be related to a decrease in D2 pituitary levels [35].

This data opens an issue on the reliability of simple TSH level measurement in the assessment of hT severity in the elderly. In particular, the same extent of TSH rise could denote a deeper state of hT in older than younger patients. Therefore, in these cases it may be helpful to take into account other parameters including FT3 and FT4 levels, FT3/FT4 ratio, TSH velocity (i.e., rate of TSH increase over time), and the clinical features of patients.

The second evidence shown by the study was the marked relationship between daily LT4 dose pro Kg taken by the patient before LT4 withdrawal and the level of TSH reached, after a month of hT, on the RAI administration day. We observed this direct relationship: the higher the dose pro Kg/day of LT4 assumed during treatment, the higher the TSH value achieved after LT4 withdrawal. Almost all factors tested, including the TSH_time0 level (i.e., TSH levels just before the start of LT4 withdrawal), Tg, TgAb, and creatinine did not affect this relationship. Furthermore, it is necessary to highlight that this evidence derives from patients subjected by default to therapy with a fixed dose of 100 µgr/day of LT4 from the day after thyroidectomy, regardless of the individual TSH target, weight, and age. To our knowledge, this is the first report about this correlation. It is reasonable to hypothesize a rebound effect of the HPta that is more powerful under a higher dose pro Kg/day of LT4 taken during hormonal replacement therapy. A similar mechanism has already been described for the variation of plasma prolactin levels in response to discontinuation of dopamine infusion [36]. This finding opens new research scenarios about the understanding of mechanisms that regulate TSH secretion, the improvement of thyroid hormone replacement therapy and clinical outcomes of hT patients. In addition to these two main evidences achieved by univariate and multivariate regression analyses, we have to also report a role for metabolic and inflammatory conditions in determining HPta. In fact, the best fit model that explains TSH_time1 variability includes BMI and SIRI. Overweight and obesity and/or progressive increase in SIRI levels were associated with lower TSH levels reached after a month of hT. These data suggest a negative interference of these parameters on pituitary secretion and enzymatic activity of deiodinases. The findings regarding obese and older patients might seem opposite to the well-recognized evidence that the normal range of TSH in these subjects is usually higher than the other [37, 38]. However, in our study we investigated which factors affect HPta and how they affect it. Furthermore, the cited evidence raised from cohorts of subjects with thyroid gland and without known thyroid diseases, instead we studied patients who underwent total thyroidectomy. Therefore, the negative effect of obesity and/or age on TSH_time1 levels opens new issues about thyroid replacement therapy in thyroidectomized patients.

Anyway, the interplay between HPT axis, inflammatory response, and nutritional status of patients has not yet been clarified [39] and it may require further investigations. Indeed, it is widely accepted that inflammation is influenced by several factors such as sex, BMI, stress conditions, and hT itself, and, thereafter, we may hypothesize a palindromic relationship between inflammation and hT [27, 29].

Finally, smoking showed a high estimated effect on TSH, but it did not reach statistical significance in any regression analysis, probably due to the very small percentage of smokers in our sample.

The strengths and limitations of the present study should be addressed. The study design was retrospective and no laboratory evaluation of the deiodinase activity of these patients was available. Furthermore, any interference on HPTa caused by various comorbidities such as heart disease, autoimmune diseases, and renal or hepatic failure were not evaluated given the risk of suspending LT4 therapy in these patients. On the other hand, this aspect confirm that a rigorous selection was performed, and it allowed us to analyse a very reliable population in a standardised overt hT. Moreover, all patients were treated with the same institutional protocol and tested by the same central laboratory; finally, these aspects have not yet been investigated in the literature.

## Conclusions

To date, TSH is considered the mainstay in the evaluation of thyroid hormonal status and this study highlights new factors and mechanisms that influence its levels. Ageing progressively reduces HPta; therefore, clinicians must keep this aspect in mind along with the level of TSH when assessing the severity of hT, mainly in the elderly. Furthermore, to our knowledge, we report for the first time a rebound effect of HPta, determined by the daily µgr pro Kg of LT4 taken by patients with hT. The inflammation and metabolic status of the patients also play a role in this phenomenon.

## Electronic supplementary material

Below is the link to the electronic supplementary material.

Supplementary Material 1

## Data Availability

Database generated during and/or analyzed during the current study is available from the corresponding author on reasonable request.
